# Unraveling the triad of immunotherapy, tumor microenvironment, and skeletal muscle biomechanics in oncology

**DOI:** 10.3389/fimmu.2025.1572821

**Published:** 2025-04-02

**Authors:** Shuang Ma, Ying Lu, Shang Sui, Jia-shuo Yang, Bing-bing Fu, Pei-xin Tan, Yicheng Chai, Jiaqi Lv, Lingyu Kong, Xiaolin Wu, Yi-bo Gao, Tao Yan

**Affiliations:** ^1^ School of Information Science and Engineering, Shenyang Ligong University, Shenyang, China; ^2^ St. John’s Kilmarnock School, Breslau, ON, Canada; ^3^ Department of Anesthesiology, National Cancer Center/National Clinical Research Center for Cancer/Cancer Hospital, Chinese Academy of Medical Sciences and Peking Union Medical College, Beijing, China; ^4^ School of Mathematics and Statistics, Liaoning University, Shenyang, China; ^5^ Department of Oral and Maxillofacial Surgery, Taikang Bybo Dental, Beijing, China

**Keywords:** tumor microenvironment, immunotherapy, immune checkpoint inhibitors, immune niches, skeletal muscle biomechanics

## Abstract

The intricate interaction between skeletal muscle biomechanics, the tumor microenvironment, and immunotherapy constitutes a pivotal research focus oncology. This work provides a comprehensive review of methodologies for evaluating skeletal muscle biomechanics, including handheld dynamometry, advanced imaging techniques, electrical impedance myography, elastography, and single-fiber experiments to assess muscle quality and performance. Furthermore, it elucidates the mechanisms, applications, and limitations of various immunotherapy modalities, including immune checkpoint inhibitors, adoptive cell therapy, cancer vaccines, and combined chemoimmunotherapy, while examining their effects on skeletal muscle function and systemic immune responses. Key findings indicate that although immunotherapy is effective in augmenting antitumor immunity, it frequently induces muscle-related adverse effects such as weakness, fatigue, or damage, primarily mediated by cytokine release and immune activation. This work underscores the significance of immune niches within the tumor microenvironment in influencing treatment outcomes and proposes strategies to optimize therapy through personalized regimens and combinatorial approaches. This review highlights the need for further research on the formation of immune niches and interactions muscle-tumor. Our work is crucial for advancing the efficacy of immunotherapy, reducing adverse effects, and ultimately improving survival rates and quality of life of patients with cancer.

## 1 Introduction

Cancer remains a major global public health concern and poses a severe threat to human health (1). Chemotherapy has long been the cornerstone of cancer treatment, plays a crucial role in inhibiting tumor cell growth (2). However, despite its anticancer effects, it also induces a series of significant side effects, among which the adverse impact on skeletal muscle function has increasingly become a research focus. Numerous clinical studies have shown that approximately 30–50% of cancer patients experience a significant decline in skeletal muscle function after undergoing chemotherapy, with an even higher proportion among elderly patients (3). This decline is primarily manifested as a reduction in muscle mass, primarily due to the direct toxic effects of chemotherapy drugs on muscle tissue, a phenomenon unrelated to the tumor response to treatment (4). The loss of muscle mass not only severely weakens patients’ physical mobility and quality of daily life but may also lead to decreased chemotherapy tolerance, exacerbated drug toxicity reactions, and even a negative impact on overall survival rates. For example, studies on chemotherapy in patients with advanced lung cancer have demonstrated a strong correlation between post-chemotherapy muscle loss and poor treatment outcomes (3). Patients with significant muscle loss exhibit substantially lower hemoglobin levels and a markedly increased risk of disease progression (5). Similarly, in patients with metastatic colorectal cancer, the muscle area decreased by an average of 6.1% during chemotherapy. Among patients with a muscle reduction ≥ 9%, the survival rates were significantly lower than those who experienced less muscle loss.

Recently, immunotherapy has emerged as a promising strategy for cancer treatment. Immune checkpoint inhibitors (ICIs) block inhibitory receptors such as CTLA-4, PD-1, and PD-L1 on immune cells, effectively activating the immune system to attack tumor cells (6). These agents have demonstrated remarkable efficacy in treating melanoma, lung cancer, and various other malignancies (7). Conversely, adoptive cell immunotherapy entails harvesting immune cells (e.g., T cells or NK cells) from a patient or donor, expanding and modifying them *in vitro*, and reinfusing them to enhance antitumor immunity. This approach has led to groundbreaking progress in the treatment of hematologic malignancies (8). Tumor vaccines aim to stimulate the body’s specific antitumor immune response (9). Some vaccines have shown significant success in preventing cervical cancer, and their potential applications in cancer treatment are being explored in clinical trials (10). However, while these immunotherapies effectively combat tumors, they may also affect skeletal muscle to varying degrees. For example, immune checkpoint inhibitors can cause immune-related adverse events affecting the musculoskeletal system; adoptive cell immunotherapy may trigger cytokine release syndrome, disrupting muscle cell metabolism and function; and tumor vaccines may lead to muscle fatigue, soreness, and other discomfort in some patients (6). Moreover, the integration of chemotherapy and immunotherapy is gaining prominence in the clinical practice (11). This therapeutic approach combines the cytotoxic effects of chemotherapy on tumor cells with immunotherapy immune activation of immunotherapy to synergistically enhance anticancer efficacy (12). In practice, the cytotoxicity of chemotherapeutic drugs and the immune response induced by immunotherapy can interact, further exacerbating skeletal muscle damage (13). For example, certain chemotherapeutic drugs can induce immunogenic cell death in tumors, promote immune cell activation, and intensify muscle damage (14). Moreover, the inflammatory response triggered by immunotherapy combined with the muscle toxicity of chemotherapy drugs can further aggravate muscle dysfunction (15).

Given the complex impact of immunotherapy and chemotherapy on skeletal muscle function, as well as their critical role in cancer treatment, in-depth research on their underlying mechanisms, comprehensive assessment of muscle function changes, and explore effective countermeasures (16). This study systematically examined the relationship between chemotherapy, immunotherapy, and skeletal muscle function, provided a detailed analysis of the associated mechanisms, evaluated the advantages and limitations of existing assessment methods, and discussed future research directions (17). The ultimate goal is to establish a solid theoretical foundation and practical guidance for protecting and improving skeletal muscle function during cancer treatment, thereby enhancing treatment outcomes and quality of life in patients with cancer (18).

## 2 Effects of immunotherapy and chemotherapy on tumor microenvironment, muscle cells, and inflammation

### 2.1 Mechanisms of immunotherapy in the tumor microenvironment

**Figure 1:**
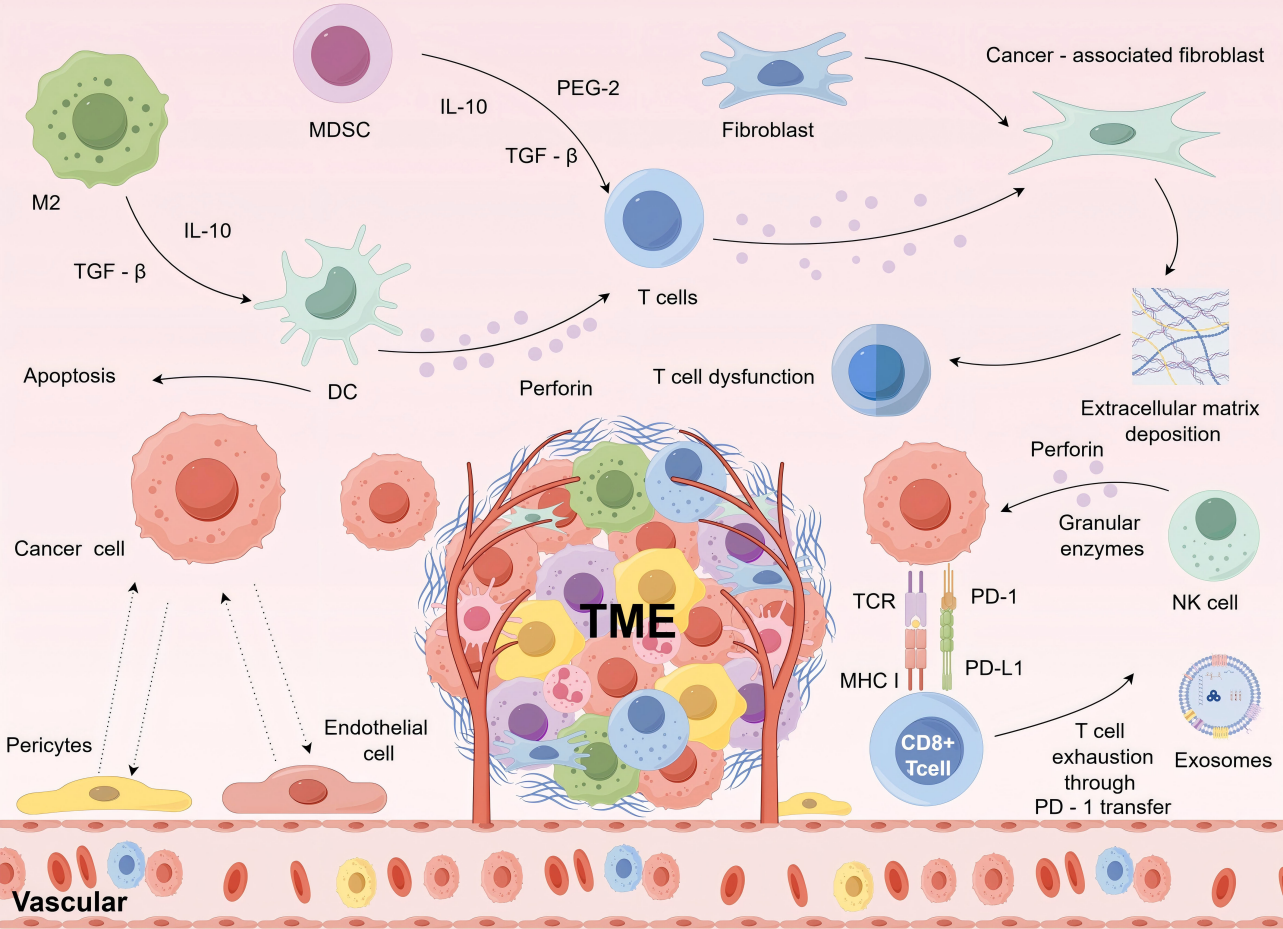
Interactions between immune cells and tumor cells in the tumor microenvironment.

The tumor microenvironment forms a complex network. This network is intricately linked to immunotherapy (19). In this microenvironment, there are various immune cells and associated cellular components. They interact dynamically with tumor cells. As shown in **Figure 1**, natural killer (NK) cells induce cytotoxicity in tumor cells by releasing perforin and granzyme, while M2-type tumor-associated macrophages (TAMs) influenced by cytokines such as transforming growth factor-beta (TGF-β) and interleukin-10 (IL-10), and interact with tumor cells to promote i (20). Dendritic cells (DCs) capture tumor antigens and activate T cells, which play a crucial role in antigen presentation within tumor immunity (21). Regulatory T cells (Tregs) secrete TGF-β and IL-10 to suppress immune responses, whereas myeloid-derived suppressor cells (MDSCs) also release these inhibitory cytokines and induce dendritic cell apoptosis. CD8+ T cells recognize major histocompatibility complex (MHC) class I molecules on the surface of tumor cells via T cell receptors (TCRs) to induce tumor cell cytotoxicity. However, interactions between programmed death receptor-1 (PD-1) and its ligand (PD-L1), as well as tumor cell-derived exosomes, lead to T-cell exhaustion. Additionally, fibroblasts can differentiate into cancer-associated fibroblasts (CAFs), contributing to extracellular matrix deposition, which further impairs T-cell function (22). This intricate interplay not only governs tumor growth, metastasis, and response to therapy but also indirectly affects the metabolism and function of normal tissues, such as skeletal muscle, through various pathways (23). Immunotherapy plays a central role in the vast ecosystem of tumor treatment. Strategies, such as ICIs targeting CTLA-4, PD-1, and PD-L1, as well as CAR-T cell therapy, aim to reprogram the immune system to better recognize and eliminate tumor cells (24). In the tumor microenvironment, immune cells undergo significant alterations (25). The activation, proliferation, and differentiation of T cells are tightly regulated, while macrophages, under tumor-derived signals, polarize into proinflammatory M1 or anti-inflammatory M2 phenotypes, dynamically shifting their cytokine production profile (26). IL-6 and TNF-α have intricate roles in polarization of immune cells.IL-6 enhances the inflammatory response by activating the STAT3 signaling pathway to promote Th17 cell differentiation and inhibit Treg cell generation.TNF-α, working together with IL-6, enhances Th17 cell polarization, worsening the immune inflammatory condition (27). This combined effect in the tumor microenvironment can contribute to immune cell dysfunction and help tumor cells evade the immune system.Conversely, in specific situations, IL-6 and TNF-α can show opposing effects. For example, TNF-α often promotes macrophages to polarize into the M1 phenotype, which has anti-tumor properties.Nonetheless, elevated levels of IL-6 might prevent M1 macrophages from polarizing and encourage their transformation into the M2 phenotype, which supports tumor growth. An imbalance in this opposing effect could affect the ability of immune cells to fight tumors within the tumor microenvironment (28). Cytokines such as IFN-γ and TNF-α travel through the bloodstream or paracrine pathways to reach skeletal muscle cells. IFN-γ binds to receptors on skeletal muscle cell surfaces, triggering the JAK-STAT signaling pathway. During this process, phosphorylated STAT proteins translocate into the nucleus, bind to the promoter regions of specific genes, and upregulate the expression of proteases, such as caspase-3, which degrade muscle proteins. In the JAK-STAT pathway, IFN-γ activates receptor-associated JAK kinases when it binds to its receptor (29).These JAK kinases have tyrosine kinase activity and, when activated, add phosphate groups to specific tyrosine residues on the receptor.The phosphorylated tyrosine residues act as docking sites for STAT proteins, which use their SH2 domains to bind to these residues and are then phosphorylated by JAK kinases. Phosphorylated STAT proteins form dimers, changing their shape and allowing them to penetrate the nucleus. After entering the nucleus, the dimerized STAT proteins attach to specific DNA sequences located in the promoter regions of target genes, including the Gamma Interferon Activation Sequence (GAS), which attracts transcription-related cofactors like RNA polymerase to start downstream gene transcription, thus controlling gene expression and affecting cellular functions (30). This disrupts the balance between muscle protein synthesis and degradation, ultimately leading to muscle atrophy. TNF-α also interferes with the insulin signaling pathways. Under normal conditions, insulin binds to its receptor and activates insulin receptor substrate (IRS) proteins, initiating the PI3K-AKT pathway, which promotes glucose uptake in muscle cells (31). TNF-α activates the MAPK pathway, causing phosphorylation-induced inactivation of IRS proteins, blocking insulin signal transmission, and reducing glucose uptake by muscle cells, leading to metabolic dysregulation. The effects of IL-6 and TNF-α on muscles are significant (32). IL-6 can trigger the ubiquitin proteasome system in skeletal muscle cells, promoting muscle protein breakdown of muscle proteins and results in muscle wasting. Moreover, IL-6 hinders the growth and specialization of muscle satellite cells, impacting muscle repair and regeneration. TNF-α not only disrupts insulin signaling pathways affecting muscle metabolism but also works with IL-6 to boost muscle protein breakdown and hinder muscle repair. Research indicates a strong link between high serum levels of IL-6 and TNF-α in cancer patients and a decline in muscle strength and mass (33). Moreover, IL-6 and TNF-α can disrupt muscle contraction by altering calcium ion balance in muscle cells, causing fatigue and weakness. Abnormal angiogenesis and extracellular matrix remodeling within the tumor microenvironment are also closely linked to skeletal muscle alterations (34). Tumor angiogenesis is a complicated process that includes multiple angiogenic factors and signaling pathways (35). Vascular endothelial growth factor (VEGF) plays a crucial role in tumor angiogenesis, as tumor cells release significant quantities of VEGF to encourage endothelial cell growth, movement, and lumen creation, thus aiding in the development of tumor blood vessels. At the same time, the angiopoietin (Ang) family and fibroblast growth factors (FGF), among others, are also involved in this process (36). The newly developed blood vessels display irregular structures and functions, with incomplete vessel walls and heightened permeability, supplying nutrients and oxygen to tumor cells while also facilitating tumor cell metastasis. Irregularities in tumor blood vessels impact the penetration of immune cells into tumor tissues, changing the immune status of the tumor microenvironment. For example, increased vascular permeability may hinder immune cells from exiting the bloodstream, thereby diminishing their ability to destroy tumor cells. The disorganized growth of the tumor vasculature not only nourishes the tumor but also deprives the surrounding tissues of nutrients, placing skeletal muscle under ischemic and hypoxic conditions. Under such microenvironmental stress, skeletal muscle mitochondria experience oxidative phosphorylation dysfunction, reducing ATP production and shifting metabolism toward anaerobic glycolysis, leading to lactic acid accumulation (37). This disrupts the intracellular pH and ion homeostasis, ultimately impairing muscle cell metabolic functions. Additionally, when stimulated by tumor-derived signals, stromal cells such as fibroblasts secrete extracellular matrix (ECM) components including collagen and fibronectin, which undergo abnormal expression and deposition (38). Collagen, fibronectin, laminin, proteoglycans, and other components make up the ECM, a vital element of the tumor microenvironment. Through different mechanisms, tumor cells and cancer-associated fibroblasts (CAFs) can change the composition and structure of the ECM.As tumors progress, collagen fibers released by CAFs are crosslinked and remodeled, resulting in stiffer ECM. The alteration in stiffness influences the ability of tumor cells to migrate, enhancing their invasion and spread (39). Modifications in the ECM also impact immune cell function. For instance, irregular ECM can disrupt the adhesion and movement of immune cells, hindering their ability to identify and destroy tumor cells. Certain elements of the ECM can engage with receptors on immune cell surfaces, modulating their activation and cytokine release, thereby affecting the immune condition of the tumor microenvironment (40). These changes, mediated by integrin receptors, affect cytoskeletal structures and mechanotransduction signaling within skeletal muscle cells, further influencing cell migration, proliferation, and differentiation (41).Tumor blood vessels and the extracellular matrix are closely linked. Irregular tumor angiogenesis influences ECM remodeling, and alterations in the ECM can impact the stability and function of tumor blood vessels. For instance, the ECM surrounding tumor blood vessels can stabilize and support them, while an abnormal ESubstances released by tumor blood vessels can influence the production and breakdown of the ECM, while ECM components can engage with receptors on endothelial cell surfaces, impacting angiogenesis and vessel performance (40). This interaction affects not just tumor growth and metastasis but also significantly alters immune cell function within the tumor microenvironment, contributing to its complexity.

Studies on prostate cancer bone metastasis have identified BHLHE22 as a key transcription factor that is highly expressed in tumor cells (42). BHLHE22 interacts with PRMT5 to form a transcriptional complex that binds to the CSF2 promoter, initiating its transcription. CSF2, an important cytokine, recruits a large number of immunosuppressive neutrophils and monocytes into the tumor microenvironment (43). These immunosuppressive cells secrete arginase-1 (Arg-1) and other immunosuppressive factors that inhibit T-cell activation and proliferation, reduce the number of CD4+ and CD8+ T cells, and impair their function (44). This promotes bone metastasis of tumor cells and creates a strongly immunosuppressive microenvironment (45). This indicates that in prostate cancer bone metastasis, a BHLHE22-PRMT5-CSF2-mediated immunosuppressive pathway exists, which interacts with immune cells in the tumor microenvironment and jointly influences tumor progression and metastasis (46). This further exacerbates its impact on normal physiological functions, including potential interference with skeletal muscle metabolism and function (47). In soft-tissue sarcomas, researchers have discovered that the transcriptional co-activator YAP1 plays a significant role in tumor cells. YAP1 promotes the deposition of collagen VI (COLVI) in the tumor microenvironment (48). COLVI interacts with collagen I (COLI) to remodel the extracellular matrix (ECM) (49). Specifically, COLVI directly modifies the architecture of COLI fibers, altering their physical properties and subsequently affecting the function of CD8+ T cells in the tumor microenvironment (50). COLVI induces CD8+ T-cell dysfunction, characterized by upregulation of inhibitory receptor expression, decreased proliferation, and reduced cytotoxic function (51). In contrast, COLI enhances CD8 + T-cell function and serves as a tumor suppressor to some extent (52). This discovery reveals that in soft tissue sarcomas, tumor cells influence immune cell function by regulating ECM components, thereby shaping a tumor-friendly immune escape microenvironment. Such alterations in the tumor microenvironment (TME) may indirectly affect the microenvironment of skeletal muscle cells by affecting local nutrient transport and metabolic waste clearance, potentially influencing skeletal muscle metabolism and function (53). Researchers constructed various genetically engineered mouse models of multiple myeloma and found that the MAPK-MYC pathway plays a critical role in disease progression (54).The activation of MYC correlates with tumor progression rate and affects immune cell infiltration and function within the tumor microenvironment. In rapidly progressing models, studies have identified a high prevalence of activated/exhausted CD8 + T cells and a reduced population of immunosuppressive regulatory T cells (Tregs). In slow progressing models, they found lower CD8+ T-cell infiltration and more Tregs, which suppressed immune responses (55). Single-cell transcriptomics and functional experiments demonstrated that the CD8+/Treg ratio could serve as an important predictor ICB therapy response. In untreated smoldering multiple myeloma patients, a high CD8+/Treg ratio is associated with early disease progression (56). In patients with newly diagnosed multiple myeloma patients undergoing Len/Dex treatment, this ratio was correlated with early relapse (57). In ICB-resistant multiple myeloma models, increasing CD8+ T-cell cytotoxicity or depleting Tregs reverses immune therapy resistance and prolongs disease control (58). These findings indicate that in multiple myeloma, the genetic characteristics of tumor cells and their interaction with immune cells in the tumor microenvironment jointly determine disease progression and response to immunotherapy (59). This alteration in the immune microenvironment may also indirectly influence skeletal muscle physiology by modulating the skeletal muscle cell energy metabolism and protein synthesis (60).There are significant commonalities in the interactions between tumor cells and immune cells among these cancers. Tregs play a role in the immunosuppressive process in prostate cancer, soft tissue sarcomas, and multiple myeloma (61). In bone metastasis of prostate cancer, Tregs secrete inhibitory cytokines that can suppress T cell activation and proliferation. Tregs can also dampen the immune response enabling tumor cells to escape immune surveillance. In multiple myeloma, variations in Treg quantity and activity are closely associated with disease progression. Simultaneously, CD8+ T cells, which are crucial effector cells in anti-tumor immunity, have a significant impact on these cancers. When functioning properly, they can identify and destroy tumor cells, but their abilities are suppressed by the tumor microenvironment. For instance, modifications in the tumor microenvironment of soft tissue sarcomas can cause CD8+ T cells to function less effectively (53). The progression of multiple myeloma and the effectiveness of immunotherapy are also impacted by the infiltration and functional status of CD8+ T cells. The key regulatory molecules and signaling pathways vary noticeably between different types of cancer. In the context of prostate cancer, BHLHE22 and PRMT5 assemble a transcriptional complex that initiates CSF2 transcription, which then attracts many immunosuppressive neutrophils and monocytes, promoting the tumor’s metastasis to the bones. In soft tissue sarcomas, YAP1 is a significant transcriptional co-activator. It encourages collagen VI deposition, alters the extracellular matrix, and specifically hinders CD8+ T cell functions, fostering an immune-escape environment for tumor cells (53). The regulation of multiple myeloma is mainly through the MAPK-MYC pathway. The activation of this pathway influences immune cell infiltration and function, with the CD8+ T cell to Treg ratio being crucial for disease progression and immunotherapy response. In skeletal muscle injury and regeneration, regulatory T cells (Tregs) are essential for modulating macrophage polarization, promoting muscle satellite cell proliferation and differentiation, and suppressing excessive inflammation (62). Tregs facilitate the conversion of M1-type (proinflammatory) macrophages into M2-type (anti-inflammatory) macrophages, which, in turn, secrete growth factors and cytokines like TGF-β, promoting muscle repair and regeneration (63). Tregs secrete amphiregulin (Areg), which acts directly on muscle satellite cells, stimulating their proliferation and differentiation, and thereby accelerating muscle repair (64). By inhibiting excessive inflammation, Tregs prevent further damage to skeletal muscle cells (65). In some muscular diseases, such as Duchenne muscular dystrophy (DMD), Tregs help suppress type I inflammatory responses, reduce muscle damage and inflammation, and slow disease progression (66). This suggests that Tregs play an essential role in maintaining skeletal muscle homeostasis and in promoting injury repair (67). Their proper function may have potential therapeutic applications in mitigating muscle-related side effects during cancer treatment, such as reducing chemotherapy- or immunotherapy-induced muscle atrophy or dysfunction (67).

### 2.2 Effects of immunotherapy and immunochemotherapy on muscles

With the widespread application of immunotherapy in clinical oncology, its combination with chemotherapy has become increasingly common, with the aim of merging the cytotoxic effects of chemotherapy on tumor cells with immunotherapy immune modulation, achieving a synergistic antitumor response (68). However, while enhancing antitumor efficacy, this combination therapy also has adverse effects on skeletal muscles. Chemotherapeutic agents exert cytotoxic effects that significantly affect the skeletal muscle (6). For example, doxorubicin can enter skeletal muscle cells through passive diffusion or active transport mechanisms, intercalate into DNA, interfere with DNA replication and transcription, and induce the production of large amounts of reactive oxygen species (ROS). This damages the membranes of mitochondria and other organelles, leading to mitochondrial dysfunction, reduced ATP production, and energy crisis within the cell (69). Cisplatin primarily forms adducts with DNA, blocks DNA repair and transcription, and triggers a series of toxic reactions such as the initiation of apoptosis (70). During this process, muscle cell mitochondria release damage-associated molecular patterns (DAMPs) including mitochondrial DNA and HMGB1 (71). These molecules are recognized by pattern recognition receptors (PRRs), such as Toll-like receptors (TLRs) on innate immune cells, thereby activating the innate immune system and triggering an inflammatory response (72).

During immunotherapy, T cells are massively activated. They proliferate and differentiate into effector T cells that infiltrate tumors. At this time, immune dysregulation may occur, leading to a cytokine storm (73) The massive release of cytokines such as IFN-γ, TNF-α, and IL-6 not only attacks tumor cells but also indirectly damages skeletal muscle cells. At the molecular level, IFN-γ can activate the ubiquitin-proteasome system, upregulating the expression of proteolytic enzymes related to actin and myosin degradation and leading to their breakdown (74). Additionally, IFN-γ can induce post-translational modifications that alter the structure and function of actin and myosin, thereby altering their kinetic properties and impairing muscle contraction (74). TNF-α can interfere with calcium regulation by activating membrane calcium channels and disrupting intracellular calcium homeostasis, leading to abnormal intracellular calcium ion concentration (75). This disruption damages the excitation-contraction coupling, resulting in impaired muscle contraction and relaxation. In clinical studies, skeletal muscle function has been assessed in cancer patients undergoing immunochemotherapy (76). In a study of non-small cell lung cancer patients, muscle force began to decline significantly around the second or third week of treatment (77). Assessments using grip strength tests and lower limb muscle force measurements revealed that the average grip strength decreased by approximately 15-20%, whereas the maximal contraction force of the lower limbs was reduced by 20-25% (78). In terms of exercise endurance, the six-minute walk distance decreased by 100-150 meters compared to the pre-treatment levels (79). Similar findings were observed in studies on patients with breast cancer, in which progressive muscle fatigue and limited physical activity were noted during treatment (80). As the number of treatment cycles increases, skeletal muscle dysfunction becomes more pronounced, characterized by muscle atrophy and a continuous decline in muscle strength (81). These findings further confirm the adverse effects of immunochemotherapy on skeletal muscle, severely affecting patients’ quality of life and treatment tolerance (82). In the realm of clinical practice, achieving a balance between effective therapy and skeletal muscle protection is highly important for patients. Prior to initiating immunotherapy, it is essential to conduct a thorough evaluation of patients, which should encompass detailed tests of muscle tests, a review of medical history (with a focus on muscle-related conditions), and assessments of physical health (83). Individualized treatment plans should be developed based on the evaluation results. In cases of patients with compromised muscle function or muscle disorders, the dosage of immunotherapy drugs may be adjusted, or drugs that have a lesser effect on muscles can be selected. Throughout the treatment, consistently check the patients’ muscle function indicators, including muscle strength and endurance, while also keeping an eye on relevant serum markers like creatine kinase (84).

### 2.3 Effect of drugs on muscle cells

Chemotherapeutic agents widely used in cancer treatment include cyclophosphamide, doxorubicin (DOX), and 5-fluorouracil (5-FU), all of which may alter muscle cell function at their final destination, including skeletal muscle contraction-relaxation properties (85). As an alkylating agent, cyclophosphamide acts on the bone marrow, bladder, lungs, and heart, and prolongs muscle paralysis through pseudocholinesterase inhibition. DOX, apart from its cardiotoxic effects, may induce muscle dysfunction, tending to cause persistent fatigue and weakness even after treatment (86). Drugs such as DOX can cause oxidative stress, resulting in increased ROS levels and disturbance of the redox balance in the muscle cells (87). Oxidative stress ultimately leads to mitochondrial damage, resulting in mitochondrial dysfunction (47). In this regard, energy metabolism and calcium homeostasis in the muscle cells can be disrupted. In addition, chemotherapy can induce structural and functional changes in the mitochondria, including swelling and rupture, vacuolization of the sarcoplasmic reticulum, inhibition of ATPase, and increased intracellular calcium concentrations, thus interfering with contraction and relaxation (88). As a result, metabolic pathways are progressively disturbed, thereby producing less ATP necessary for muscle contraction and leading to decreased strength and endurance (89).

Clinical trials that considered metabolic changes after chemotherapy in cancer patients showed significant losses in both muscle mass and strength (90). For example, trials in post-gastrectomy patients have suggested that adjuvant chemotherapy might further deteriorate lean body mass loss, which again negatively affects the patient’s functional ability, quality of life, drug efficacy, and recovery (91). The effects of chemotherapy on skeletal muscle depend on the mode of administration, dose, and patient variables (92). For example, studies conducted on healthy mice treated with single or multiple doses of docetaxel did not show significant changes in muscle strength, implying that additional research is required to explain the impact of chemotherapy on the muscles (93).

Chemotherapeutic drugs can affect various metabolic pathways that directly or indirectly affect skeletal muscle function. For instance, the CAF regimen widely used in breast cancer, which includes cyclophosphamide, doxorubicin, and 5-fluorouracil, can induce muscle catabolism through oxidative stress associated with DOX metabolism in both liver and muscle tissues (94). Similarly, S-1, which is widely used in adjuvant therapy for gastric cancer, facilitates the loss of muscle mass through mechanisms that could implicate toxic metabolites arising during its metabolism, thereby acting directly on the muscle. The rate of drug clearance influences the duration and extent of exposure of the skeletal muscle to chemotherapy. Therefore, individual variability in genetic and physiological factors leads to variability in clearance (95). Therefore, similar regimens may affect the skeletal muscle of different patients (96). Studies conducted on this issue have estimated a decline in clearance following chemotherapy, which prolongs the retention time of the drug and enhances catabolism and dysfunction. Chemotherapeutic drugs are primarily cleared by metabolic enzymes (3). These enzymes exhibit different activities and expression levels, which are influenced by chemotherapeutic drugs (97). Recently, new targeted chemotherapy drugs have been developed, showing distinct benefits in precise cancer therapy, but their possible effects on muscles have increasingly become a focus. PARP inhibitors work against cancer by blocking poly(ADP-ribose) polymerase (PARP), which in turn disrupts the DNA repair process in cancer cells. Research indicates that PARP inhibitors might influence the energy metabolism within muscle cells (98). In studies with mice, prolonged use of PARP inhibitors leads to reduced ATP levels in muscle tissue, which impacts normal muscle contraction. PARP inhibitors might disrupt the function of mitochondria in muscle cells, affecting the respiratory chain and leading to decreased ATP production. PARP inhibitors might influence the redox equilibrium in muscle cells, resulting in the buildup of reactive oxygen species (ROS), which can initiate oxidative stress responses and harm muscle cells. Antibody-drug conjugates (ADCs) are a new type of targeted chemotherapy that combines monoclonal antibodies, cytotoxic agents, and linkers to accurately deliver toxic drugs to cancer cells (99). Although ADCs improve anticancer effectiveness and minimize toxicity to healthy tissues, their effects on muscles should not be ignored. Clinical research has shown that some patients receiving ADCs report symptoms like muscle weakness and fatigue. Small-molecule inhibitors aimed at the epidermal growth factor receptor (EGFR) have been documented to potentially impact the proliferation and differentiation of muscle cells, in addition to inhibiting tumor cell growth. Laboratory studies have indicated that EGFR inhibitors might disrupt the EGFR signaling pathway in muscle satellite cells, hindering the activation and differentiation of satellite cells, which impacts the muscles’ ability to repair and regenerate (100). This results in a complex regulatory feedback mechanism. Some studies suggest that chemotherapy drugs induce or inhibit the activity of certain enzymes that affect drug metabolism and clearance, and may also alter muscle biomechanics (101).

### 2.4 Immune system and inflammatory response

**Figure 2:**
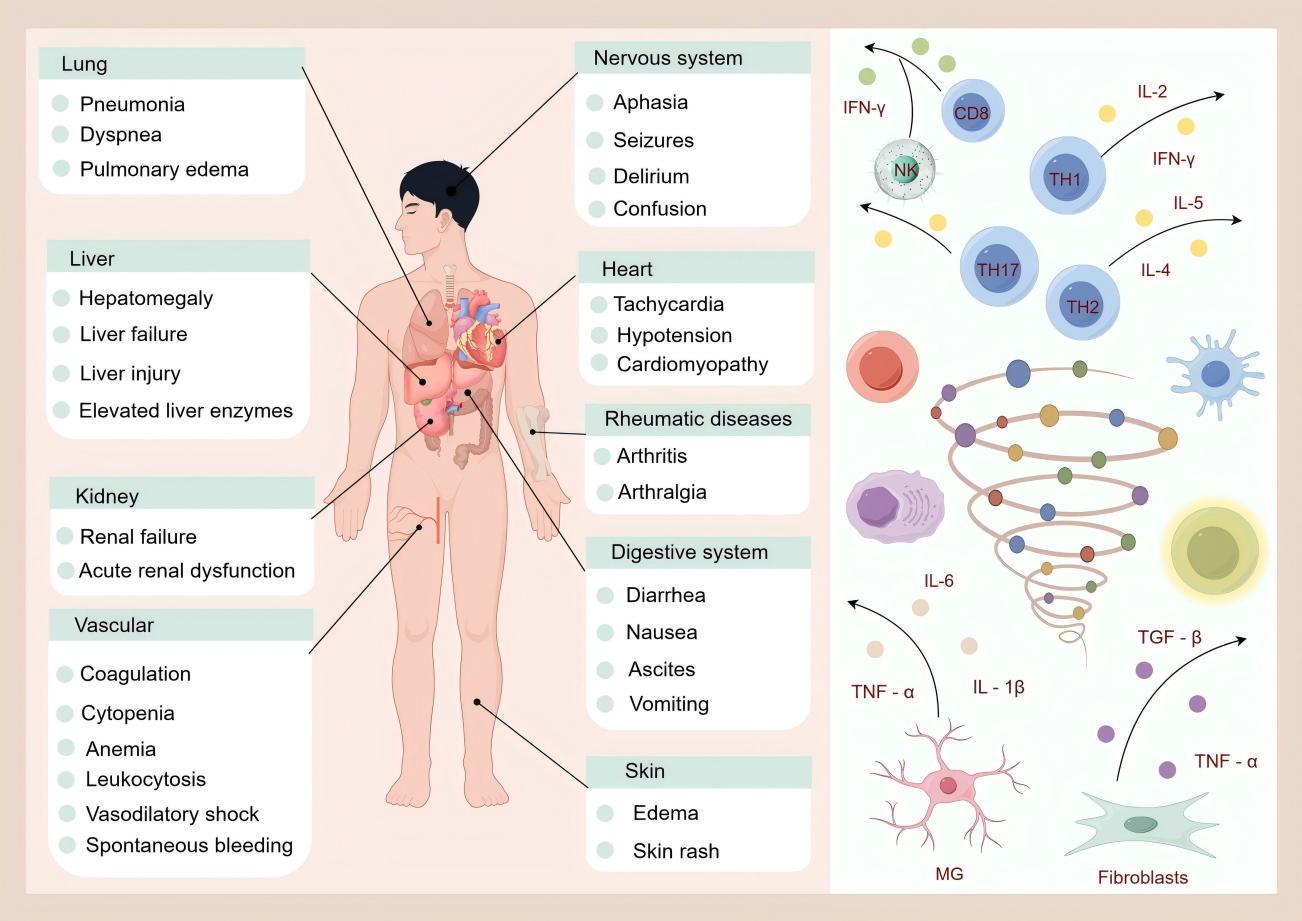
Cytokine storm and its clinical manifestations.

During the progression of various diseases, the immune system and inflammatory responses are intricately intertwined and mutually influential, profoundly altering disease trajectories and outcomes (102). These processes also play a significant role in skeletal muscle function, with cytokine storms often serving as the key factors (**Figure 2**). Cytokine storms present with a wide range of clinical manifestations, including pneumonia, respiratory distress, and pulmonary edema in the lungs; hepatomegaly, liver failure, liver injury, and elevated liver enzymes in the liver; kidney failure and acute renal dysfunction in the kidneys; coagulation abnormalities, cytopenia, anemia, leukocytosis, vasodilatory shock, and spontaneous bleeding in the vascular system; aphasia, seizures, delirium, and altered consciousness in the nervous system; tachycardia, hypotension, and cardiomyopathy in the heart; arthritis and joint pain associated with rheumatic diseases; diarrhea, nausea, ascites, and vomiting in the digestive system; and edema and rashes in the skin. In fibrotic diseases, excessive extracellular matrix (ECM) deposition and impaired degradation are central pathological features. Inflammatory responses trigger the release of cytokines, including TGF-β, TNF-α, and the IL family, which activate fibroblasts, promote epithelial-mesenchymal transition (EMT), endothelial-mesenchymal transition (EndoMT), and mesothelial-mesenchymal transition (MMT), and promote the excessive generation of myofibroblasts. This results in excessive ECM production, which disrupts the skeletal muscle microenvironment (103).

In systemic autoimmune fibrotic diseases, such as systemic sclerosis (SSc), excessive autoantibody production and abnormal activation of immune cells(e.g., T cells, B cells, and macrophages) drive tissue infiltration. Imbalances in T-cell subsets, such as increased secretion of Th2 cytokines that promote fibroblast activation and extracellular matrix (ECM) deposition, and Th17 cells producing IL-17 to amplify inflammation, induce tissue damage, suppress regulatory T (Treg) cell function, and exacerbate autoimmune responses and fibrosis (104). Macrophages polarize into the M1 and M2 subtypes. M1 macrophages drive early inflammation, whereas M2 macrophages secrete TGF-β and PDGF in the later fibrotic stages, promoting myofibroblast differentiation and ECM synthesis, thereby accelerating fibrosis (105). In liver fibrosis, activated macrophages undergo phenotypic shifts and release cytokines that activate stellate cells, thereby inducing excessive ECM deposition (106). Systemic immune dysregulation and chronic inflammation further impair skeletal muscle protein metabolism and function (107). During tumor progression, the tumor microenvironment (TME) maintains a chronic inflammatory state (19). Immune cells such as tumor-associated macrophages (TAMs) and neutrophils secrete cytokines that promote tumor cell proliferation and migration (108). TAMs polarized to the M2 phenotype release TGF-β and VEGF, thereby suppressing antitumor immunity, whereas tumor cells produce immunosuppressive molecules such as PD-L1 to deactivate T cells and facilitate immune escape (109). For example, chronic intestinal inflammation in colorectal cancer increases tumor risk and immunosuppressive TME weaken immune surveillance. During cancer therapy, particularly immunotherapy, systemic immune hyperactivation may trigger a cytokine storm. Overproduction of cytokines such as IFN-γ, TNF-α, and IL-6 indirectly damages skeletal muscle cells; IFN-γ activates the ubiquitin-proteasome system, degrading actin and myosin, whereas TNF-α disrupts excitation-contraction coupling and impairs muscle function (15). Cytokine storms also elevate serum levels of TNF-α and IL-6, inducing protein breakdown, suppressing synthesis, and hindering muscle fiber regeneration, thereby forming a vicious cycle (110). Chemotherapeutic drugs, such as S-1, further activate immune cells, alter cytokine profiles, and disrupt skeletal muscle metabolism. Prolonged inflammation exacerbates muscle fiber damage. Although immunotherapies (e.g., checkpoint inhibitors and CAR-T) show efficacy, adverse effects, such as cytokine storms (common in CAR-T therapy), may worsen skeletal muscle injury and compromise treatment outcomes. In neurodegenerative diseases such as Alzheimer’s disease (AD), activated microglia initiate neuroinflammation by releasing cytokines (IL-1β, IL-6, and TNF-α), leading to neuronal damage, Aβ plaque accumulation, and tau hyperphosphorylation. Peripheral T-cell infiltration into the brain may alter microglial function and Aβ metabolism, thereby accelerating disease progression. Neurological disorders affect skeletal muscle via neuromuscular junctions or neuroendocrine pathways, causing atrophy and weakness (111). Cytokine storms can amplify neuroinflammation and muscle damage, thereby complicating disease management. In osteoporosis, bidirectional interactions exist between the immune system and skeletal muscles (112). Postmenopausal osteoporosis (PMOP), age-related osteoporosis, and diabetic osteoporosis involve estrogen deficiency, aging, and hyperglycemia, which alter immune cell function, elevate proinflammatory cytokines, stimulate osteoclastogenesis, and disrupt T-cell balance (112). Altered bone structure and biomechanics in osteoporosis modify mechanical loading on muscles, leading to long-term atrophy and functional decline. Muscle-derived factors also regulate bone metabolism by interacting with the immune and inflammatory pathways. Cytokine storms may further destabilize immune homeostasis, intensify inflammation, and accelerate bone loss and muscle damage. Immune and inflammatory responses are intricately linked to skeletal muscle in multiple diseases. Cytokine storms exacerbate disease complexity and severity and negatively affect muscle function. A deeper understanding of these interactions is critical for developing effective therapies and improving patient prognosis (113).

## 3 Methods and tools for assessing skeletal muscle biomechanics

### 3.1 Methods for evaluating skeletal muscle biomechanics

#### 3.1.1 Assessment of muscle force

Initial screening can be performed using Manual Muscle Testing (MMT), which is convenient, but highly subjective. To obtain more objective data, a hand-held dynamometer (HHD) can be used to measure maximal voluntary isometric strength such as knee extension muscular force (114). Additionally, leg power devices can be used to assess lower limb muscular force by providing quantifiable data and highly reliable results. To study variations in muscle force with speed, a length-tension instrument (ID) can accurately capture force variation curves across the full range of motion, thereby facilitating a deeper understanding of muscle function (115).

**Figure 3:**
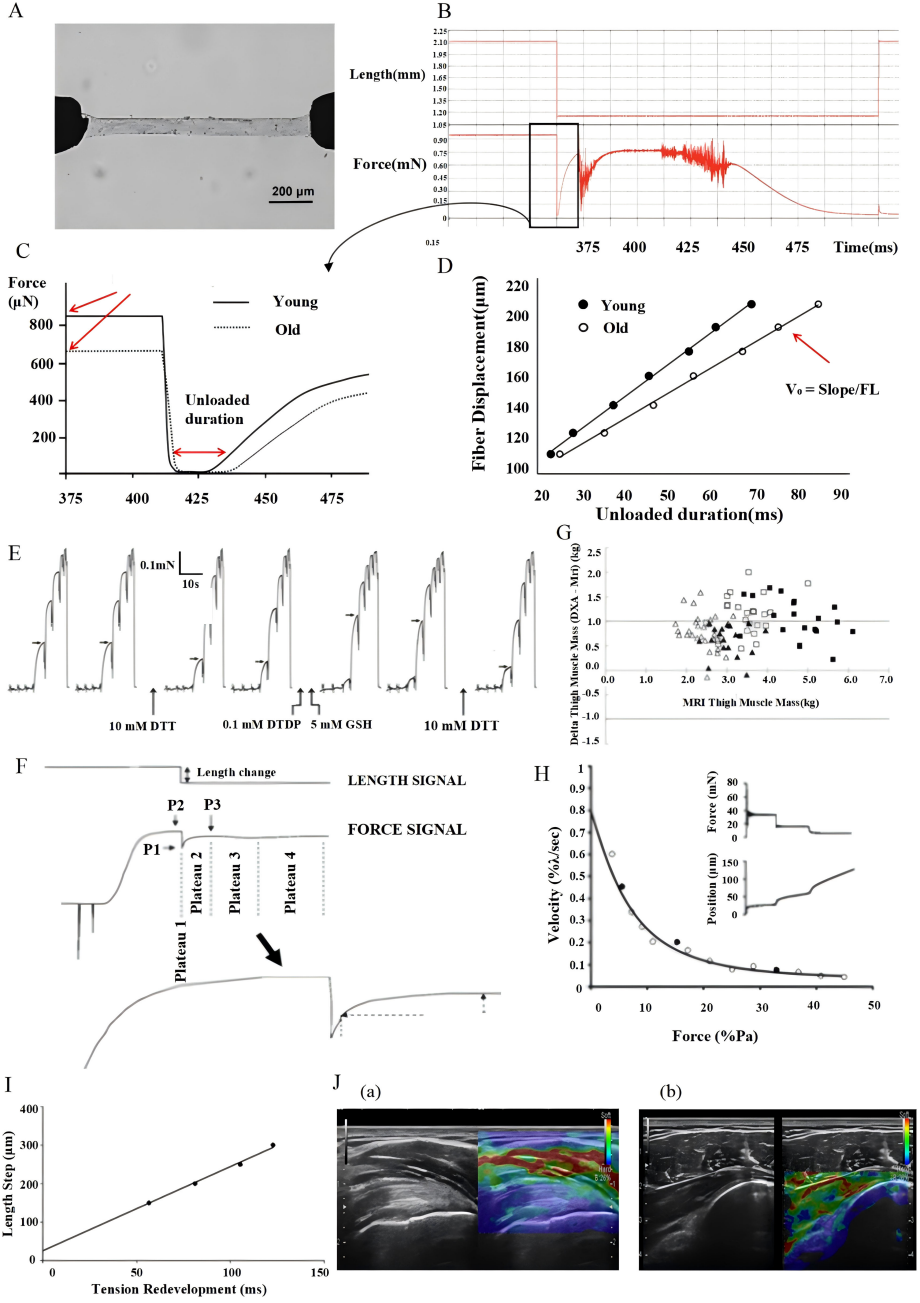
Diagram of percutaneous muscle biopsy experiment. **(A)** Image of the permeabilized fiber connected to a force transducer and servo motor. **(B)** Relaxation testing of the activation solution. **(C)** Duration of unloading after the peak force was achieved. **(D)** Unloaded shortening velocity. **(E)** Force response of vastus lateralis fibers exposed to DTT and DTDP-GSH. **(F)** Phases experienced by isometrically activated fibers during rapid length release. **(G)** Bland-Altman plot showing thigh muscle mass determined by MRI on the x-axis. **(H)** Isotonic contraction experiments, measuring force-velocity relationship. **(I)** Shortening velocity after fiber unloading. **(J)** Strain ultrasound elastography of the supraspinatus tendon (a), infraspinatus tendon, and posterior capsule (b).

In addition to direct strength measurements, other techniques such as percutaneous muscle biopsy have also been used to assess muscle biomechanical properties. Under local anesthesia, a muscle sample was extracted from the vastus lateralis of the thigh and promptly placed in a culture dish containing paraffin oil. The sample was then kept on a 10°C ice pack to preserve freshness and physiological activity. Subsequently, it underwent cutting and chemical peeling to isolate individual fiber segments, which were then treated in a relaxing solution at 4°C for 24 h to complete the chemical peeling process (116). Following treatment, the fiber segments were stored at -20°C to maintain bioactivity and structural integrity for further experimentation. On the day of the experiment, the fibers were treated in a relaxing solution containing 0.5% Brij-58 for 30 min to enhance permeability and then mounted onto an experimental apparatus (**Figure 3A**) equipped with a high-precision force sensor and a DC torque motor. This setup simulates physiological muscle contraction and relaxation by measuring the force generated during contraction or stretching, respectively. During testing, fibers were exposed to various Ca2+ concentrations to establish the “force-Ca2+ relationship,” and the data were analyzed using GraphPad Prism 6 software for Hill curve fitting to determine the pCa50 and Hill coefficients. Additionally, the impact of DTDP-GSH complexes on fiber Ca2+ sensitivity was assessed by exposing the fibers to 100μM DTDP solution for 5 min, followed by 2 min in 5 mM GSH solution, and recording the changes in pCa50 (**Figure 3E**) (117). Relaxation tests in activation solutions containing specific Ca2+ concentrations involved introducing a rapid relaxation step via the servomotor once the peak steady-state force was achieved (**Figure 3B**), simulating mechanical changes in the muscle during rapid contraction or extension, and recording data at the peak force (**Figure 3C**). The force response in the rapid release phase was divided into four stages (**Figure 3F**) to evaluate instantaneous response and recovery capabilities. The instantaneous stiffness and time required for the force to reach half-maximal (t1/2) were calculated to quantify the mechanical properties of the fibers (**Figure 3D**). These experiments were repeated for different relaxation lengths to ensure consistent measurement. Through this series of experiments, comprehensive data on muscle fiber mechanical properties such as instantaneous stiffness and unloaded shortening velocity were obtained, offering valuable insights into the mechanical behavior of muscle fibers (118).

#### 3.1.2 Assessment of muscle mass

A single-fiber experiment is a method for assessing muscle quality and function at the muscle-cell level. The direct measurement of the mechanical properties of individual muscle fibers eliminates the influence of the nervous system, tendons, and extracellular matrix. This method allows researchers to directly evaluate the function of myofibrillar proteins, providing a more precise understanding of the mechanical changes in muscles, which are essential indicators of muscle quality (119). MRI and DXA are two of the most valuable non-invasive imaging modalities that provide complete information on the quality and distribution of muscles (**Figure 3G**) (120). Using magnetic fields and radio waves, MRI generates highly detailed images of muscles, which allows for quantitative volume measurement and observation of internal structural changes. The technology behind DXA discrimination of fat and muscle, and the process of measuring bone mineral content in body tissues, involves emitting two X-ray beams at varying energy levels and assessing their absorption after passing through the body. These two techniques will enable determination of the effects of chemotherapy on the biomechanical properties of skeletal muscles.

#### 3.1.3 Assessment of muscle performance

Physiological and biochemical tests at the single-fiber level are powerful tools for comprehensively assessing muscle efficiency and functional status. To investigate the mechanisms underlying muscle performance, researchers have applied single-fiber techniques, that is, isolated and fixed muscle fibers with great precision. Using high-precision force sensors and fine-tuned motors, they simulated the natural state of human muscles, conducted isotonic contraction experiments to measure the force-velocity relationship, and applied the hyperbolic Hill equation to calculate the absolute and normalized powers of the fibers (**Figure 3H**) (121). Furthermore, measurements of the peak force, unloaded shortening velocity, residual force enhancement, and residual force depression have allowed researchers to unmask muscle responses to a prior contraction history. Passive elasticity measurements include the stretching of fibers of various lengths to measure muscle stiffness and elasticity. The relaxation distance was plotted based on the time of unloaded shortening for different activation and release lengths (**Figure 3I**). Finally, the peak power was calculated by measuring the force generated at the maximum contraction speed, and muscle calcium sensitivity was evaluated by changing the concentration of calcium ions. Thus, all the above-mentioned parameters provide a full understanding of the dynamics of the muscle, along with its activation efficiency (122).

#### 3.1.4 Assessment of muscle stiffness

Mechanomyography (MMG) examines dynamic muscle stiffness by measuring the natural oscillation frequency and damping ratio of muscles in response to short mechanical stimulation. Owing to its ease, low cost, and minimal dependence on technical expertise, this technique has been widely applied in clinical and research settings. Myotonometry has special applications in the assessment of muscle stiffness variations under distinct conditions of muscle contraction (123). For example, muscle stiffness can increase after eccentric exercise, which indicates the degree of muscle damage. Myotonometry can monitor and quantify these changes in muscle stiffness in real time and therefore provide an indication of the degree of muscle damage and recovery. Myotonometry demonstrated high internal consistency, ensuring stability and reproducibility during the assessment. However, some influencing factors must be considered when this device is put into practical use (124). For example, the muscle composition, length, cross-sectional area, and selection of measurement points for different subjects may affect the evaluation results. Therefore, when conducting myotonometric assessments, these variables should be strictly controlled to ensure the accuracy of evaluation results.

Shear wave elastography is an advanced non-invasive ultrasound technology with unique advantages in the measurement of biomechanical properties, especially muscle stiffness, in skeletal muscles (125). This technology is divided into two main types: static shear wave elastography (SSE) and dynamic shear wave elastography (SWE). The SSE measures the strain variation induced by external compressive pressure to provide qualitative information on tissue hardness (**Figure 3J**). SWE employs an acoustic radiation force to generate and propagate shear waves within tissues, which in turn measures the speed of propagation of shear waves to quantify the hardness of tissues. This provides much detail and objectivity in the analysis of mechanical properties. During the measurement of muscle hardness, SWE is more sensitive and accurate than SSE. SWE can directly quantify the propagation speed of shear waves, which is linearly related to tissue stiffness. Therefore, it reflects the precise mechanical status of muscles (125).

### 3.2 Tools for assessing muscle function

#### 3.2.1 Imaging technologies

**Figure 4:**
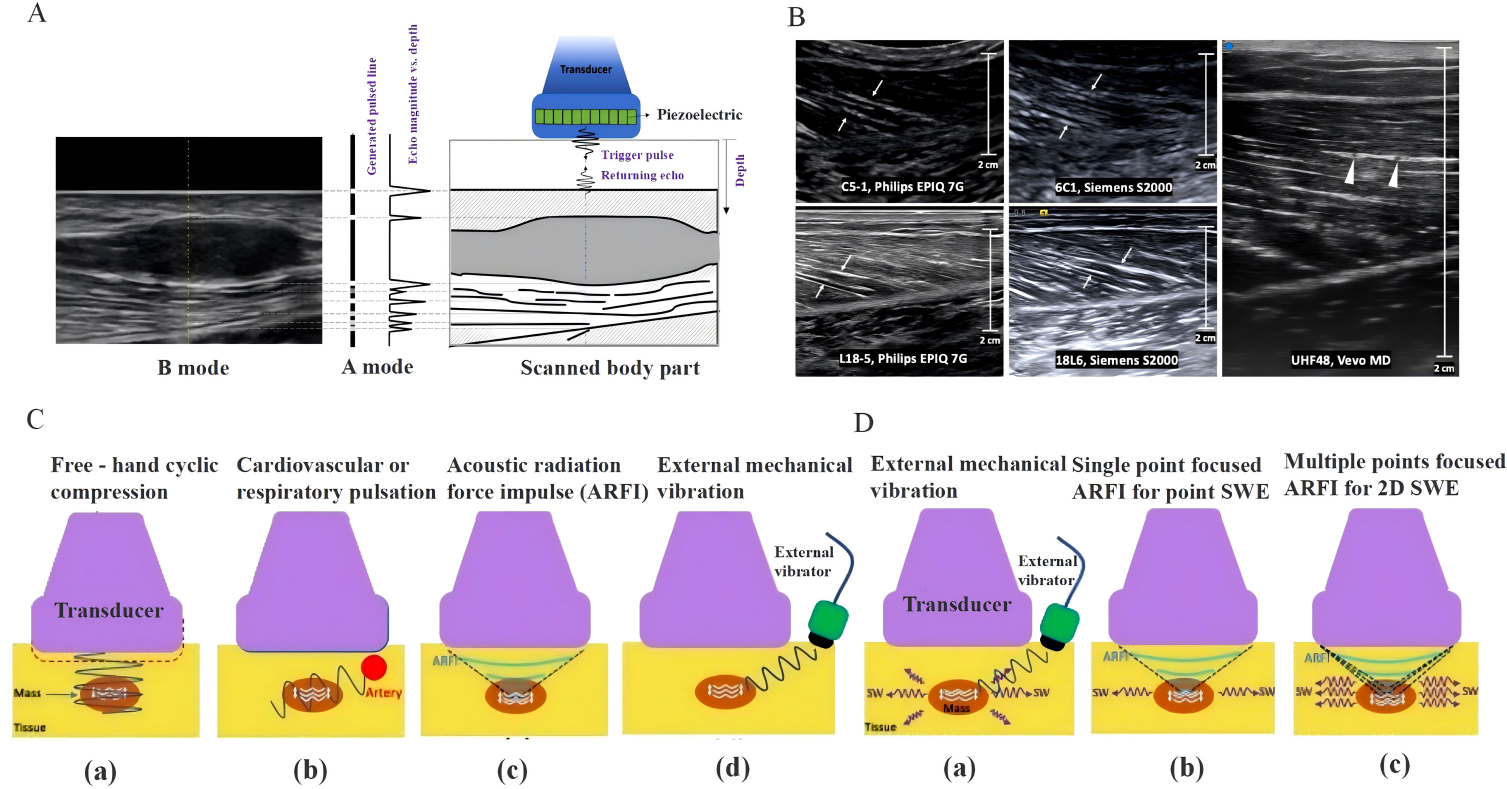
**(A)** Illustration of data generation in A- and B-mode ultrasound imaging. **(B)** Longitudinal B-mode ultrasound image of the medial gastrocnemius muscle of a healthy volunteer. **(C)** Diagram of strain ultrasound techniques, including freehand cyclic compression (a), internal organ pulsation from the heart and lungs (b), acoustic radiation force impulse (c), and external source vibration (d). **(D)** Schematic of shear-wave elastography techniques, including external mechanism vibration (a), single-point focused acoustic radiation force impulse (b), and multi-point focused acoustic radiation force impulse (c).

Imaging technologies play a critical role in evaluating the impact of chemotherapy on the biomechanical properties of skeletal muscles. These technologies enable visual observation and quantification of structural changes, fiber orientation, and tissue characteristics of the muscles (126). Ultrasound imaging techniques, including A-mode, B-mode, and M-mode, offer a relatively economical and portable method for the real-time monitoring of muscle function (**Figure 4A**). A-mode ultrasound creates images based on the relationship between echo intensity and time, is commonly used for measuring muscle thickness, and generates two-dimensional images through transducer scanning, providing a visual view of muscle fibers and connective tissues, which is highly suitable for clinical muscle analysis (**Figure 4B**). In contrast, M-mode displays echoes of moving structures and is often applied in cardiac muscle assessments. Despite the advantages of ultrasound imaging, such as being non-invasive and allowing real-time monitoring, it has a limited field of view, high operator dependency, and insufficient penetration.

Magnetic Resonance Imaging (MRI), with its high-resolution and multi-contrast imaging capabilities, plays an essential role in evaluating muscle morphology, structure, and function (127). MRI can provide detailed cross-sectional images of muscles, helping researchers understand muscle fiber types, fat infiltration, and muscle injuries, making it a powerful tool for assessing muscle mass and health (128). However, MRI is expensive, complex, and often requires prolonged cooperation, which makes it unsuitable for all patients.

Elastography assesses tissue stiffness by measuring its response to mechanical pressure, thereby providing information complementary to traditional anatomical imaging (**Figure 4C**). Strain elastography and shear wave elastography quantitatively assessed tissue stiffness through tissue displacement and shear wave propagation speed, respectively, to understand the functional changes in the muscle (**Figure 4D**) (129). Clinically, elastography is widely used for disease diagnosis in multiple organs, helping doctors determine the nature of nodules or masses by evaluating the tissue stiffness. Although elastography has the advantage of being non-invasive, it is technically complex, quantitative analysis is challenging, and is yet to be widely adopted in routine clinical examinations.

#### 3.2.2 Electrical impedance myography

Electrical impedance myography (EIM) was used to evaluate muscle function. The EIM measures muscle electrical impedance by the application of low-intensity, multi-frequency alternating current to the muscle, and provides a quick, non-invasive, and relatively inexpensive means to evaluate muscle mass and health. The technical principle of EIM is based on the impedance characteristics of the muscles to electric current. The EIM reflects the microscopic structure and functional state of the muscle by analyzing how the current propagates through the muscle (130). The advantage of this technology is that it uses low-intensity current that is harmless to the body. The testing process is quick and convenient because the current is constrained within the muscle tissue and shuns low-resistance pathways such as major blood vessels and arteries.

Unlike whole-body BIA, EIM is unaffected by individual hydration levels. In addition, the results from the EIM measurements are related to the biomechanical properties of the muscle, such as the capacity for force generation, which may make the EIM one of the most valuable tools for evaluating the impact of chemotherapy on muscles. Nevertheless, some of the advantages of the EIM include its limitations. For example, EIM depend on the skin and subcutaneous fat layer; further research is required to address these issues. However, the ability to grade the deeper muscles remains unexplored. However, EIM is a low-cost technology that is more accessible and therefore becomes an assessment tool with much value in both the clinical and research realms compared to costly imaging modalities, such as MRI (130).

## 4 Immunotherapy and tumor microenvironment in clinical oncology

Immunotherapy has become a cornerstone of cancer treatment, with the dynamic development of diverse treatment modalities presents unprecedented opportunities coupled with clinical challenges. This article systematically delineates the principal mechanisms of action characterizing contemporary immunotherapeutic interventions, evaluates their translational applications in neoplastic diseases, and critically examines persistent obstacles using proposed resolution strategies (131).

### 4.1 Immune checkpoint inhibitor

**Figure 5:**
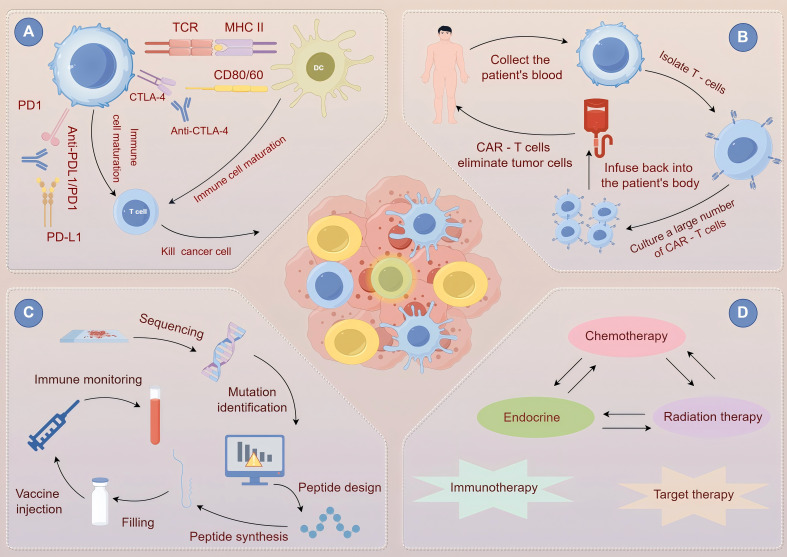
Principles and interrelationships of various cancer treatment strategies. **(A)** immune checkpoint inhibitor therapy. **(B)** Chimeric Antigen Receptor T-Cell (CAR-T) therapy. **(C)** Cancer Vaccine Therapy. **(D)** Combined Chemotherapy and Immunotherapy.

ICIs enhance immune activation by blocking inhibitory receptors, such as CTLA-4 and PD-1/PD-L1, enabling immune cells to target and attack tumor cells (**Figure 5A**) (24). To prevent the immune system from becoming overactive, immune checkpoints serve as regulatory mechanisms that maintain balance under normal circumstances. Immune checkpoints are often used by tumor cells to escape immune system attacks. ICIs inhibit molecules like CTLA-4, PD-1, or PD-L1, freeing the immune system from suppression and activating immune cells to boost the anti-tumor response (132). In cases of melanoma and non-small cell lung cancer, these inhibitors can increase patient survival and boost their quality of life. Pembrolizumab, a PD-1 inhibitor, has demonstrated significant efficacy in melanoma treatment in clinical studies. A large-scale clinical trial reported an objective response rate (ORR) of approximately 40%, with some patients experiencing a significant extension in survival and others achieving progression-free survival (PFS) exceeding five years (133). ICIs, either as monotherapy or in combination with chemotherapy, have become the standard first-line treatment for patients with advanced non-small cell lung cancer (NSCLC), especially in cases with elevated PD-L1 expression. A multicenter, randomized controlled trial found that patients receiving ICIs combined with chemotherapy had a median overall survival (OS) of approximately six months compared to those receiving chemotherapy alone. Additionally, the risk of disease progression is reduced by approximately 40%, which significantly improves patient prognosis (134). However, ICIs are not without risks and may cause immune-related adverse effects, particularly those affecting the skeletal muscle system. The cytotoxic effects of chemotherapy can damage skeletal muscle, and this damage may be exacerbated by inflammation induced by immunotherapy. For example, cyclophosphamide can penetrate muscle cells, causing DNA cross-linking damage and disrupting intracellular calcium homeostasis, thereby impairing normal muscle function (133). Simultaneously, inflammation triggered by immunotherapy leads to the release of various inflammatory cytokines, and when combined with the direct cytotoxic effects of chemotherapy, can further disrupt muscle structure and function, aggravating muscle damage. Clinically, approximately 30% of patients develop varying degrees of muscle symptoms, commonly including muscle pain, which can present as dull, stabbing, or throbbing pain, predominantly affecting the proximal limb muscles such as the shoulders and hips. Additionally, patients frequently experience muscle weakness, making basic activities, such as combing hair or standing up from a chair, difficult and severely impacting daily life. In clinical practice, a comprehensive pre-treatment evaluation of a patient’s medical history, particularly any history of autoimmune diseases or muscle disorders, along with a detailed physical examination, including muscle strength tests and joint mobility assessments, is crucial in predicting the risk of adverse reactions (135). During treatment, muscle function assessments should be conducted regularly (every 2-4 weeks), and key biomarkers, such as serum creatine kinase (CK) and lactate dehydrogenase (LDH), should be closely monitored along with careful observation of muscle symptoms. If a patient develops muscle-related adverse effects, mild cases may be managed by temporary discontinuation of ICIs and administration of nonsteroidal anti-inflammatory drugs (NSAIDs), such as ibuprofen, to alleviate pain and inflammation, while closely monitoring symptom progression. In severe cases, where muscle weakness significantly impairs mobility or CK levels rise beyond five times the normal upper limit, immediate cessation of ICI treatment is necessary and corticosteroid therapy (e.g., prednisone) should be initiated (136). Once symptoms improve, the decision to resume ICI therapy or adjust the dosage should be made based on a comprehensive evaluation of the patient’s condition.

### 4.2 Adoptive cell immunotherapy

Adoptive cell immunotherapy involves harvesting immune cells, including T cells and natural killer (NK) cells, from the patient or donor, expanding and modifying them *in vitro*, and then reinfusing them into the patient to enhance the body’s antitumor immune response (137). Various factors control the survival of immune cells in the body, with the condition of the cells being crucial. *In vitro* expansion and modification procedures can impact the expression of surface molecules and the internal signaling pathways within these cells. Taking T cells as an example, the activated costimulatory molecule CD28 can initiate a series of intracellular signal transduction pathways, promote the expression of anti-apoptoticroteins such as Bcl-2inhibit apoptosis, and prolong the survival time of T cells (138). The survival of immune cells is greatly influenced by the *in vivo* microenvironment, which contains various cytokines in the tumor microenvironment. IL-2 and IL-15 are cytokines that connect with specific receptors on immune cells, initiating pathways like JAK-STAT and delivering survival signals to these cells. Crucial interactions occur between immune cells and tumor cells, as well as with tumor-associated stromal cells. Inhibitory factors such as TGF-β, secreted by tumor cells, can impede immune cell survival. In specific scenarios, tumor-associated macrophages can affect the survival of immune cells by either cytokine secretion or direct contact. The expansion of immune cells within the body is contingent upon the combined stimulation from multiple signals. The initiation of T cell proliferation is largely due to the interaction between the T-cell receptor (TCR) and the antigen-peptide-MHC complex on tumor cells, which activates signaling pathways such as PLC-γ and Ras-MAPK (139). Following the activation of the PLC-γ pathway, there is an increase in intracellular calcium ion concentration, which activates calcineurin and facilitates the activation of the NFAT transcription factor, thereby regulating gene expression linked to cell proliferation. The Ras-MAPK pathway triggers protein kinases, encourages the expression of proteins related to the cell cycle, and allows T cells to begin proliferating. Besides the TCR signal, the costimulatory signal is essential. For instance, the interaction between CD28 and CD80/CD86 can boost the proliferation capacity of T cells. Cytokines are also crucial in the proliferation of immune cells. Immune cell proliferation can be promoted by cytokines like IL-2, IL-7, and IL-15. IL-2 engages with its receptor on immune cells, activating the JAK-STAT pathway and leading to cell growth. For immune cells to perform their roles, they must first migrate to tumor tissues, a pAdhesion molecules, including integrins like α4β1 and αLβ2, and selectins like L-selectin, are expressed on the surfaces of immune cells. Tumor tissues release chemokines such as CCL2 and CCL5. Process reliant on adhesion molecules and chemokine receptors on their surfaces (140). Chemokine receptors like CCR2 and CCR5 on immune cell surfaces bind specifically to chemokines, triggering intracellular signaling, that leads to cell polarization and movement towards higher chemokine concentrations. The relationship between immune cells and vascular endothelial cells is also highly significant. The binding of adhesion molecules on immune cells to ligands on vascular endothelial cells allows immune cells to adhere to the vascular endothelium. Afterward, immune cells move through the spaces between endothelial cells to infiltrate the tumor tissue. Chimeric Antigen Receptor T-cell (CAR-T) therapy has achieved remarkable success in the treatment of hematological malignancies (**Figure 5B**). CAR-T cell therapy is a customized form of immunotherapy that employs genetic engineering to connect a single-chain antibody targeting tumor-associated antigens with a T-cell activation domain, creating a chimeric antigen receptor (CAR). The patient’s T cells are then modified to include the gene that encodes CAR (141). These altered T cells are capable of identifying antigens on tumor cell surfaces, which activates their killing function and allows them to target tumor cells specifically. The process starts by collecting T cells from the patient, and then introducing the CAR gene into these T cells *in vitro* using retroviral or lentiviral vectors. These CAR-T cells are grown and multiplied *in vitro* to achieve a therapeutic scale. Ultimately, the patient receives the re-infused expanded CAR-T cells. These cells will identify and attach to specific antigens on the surface of tumor cells in a living organism, triggering the T cells’ killing mechanism (73). Discharge cytotoxic agents like perforin and granzymes to directly eliminate tumor cells, while also releasing cytokines to attract and activate other immune cells, thus boosting the body’s antitumor immune response. In the treatment of relapsed/refractory acute lymphoblastic leukemia (ALL), CAR-T therapy has shown a complete remission rate of 70-90% (142). A multicenter study on relapsed/refractory ALL in over 100 patients reported that approximately 75% of patients achieved complete remission, with a median remission duration of 12 months. In addition to its effectiveness in treating ALL, CAR-T cell therapy has demonstrated considerable promise in addressing other blood cancers. For example, in treating relapsed or refractory non-Hodgkin’s lymphoma, several clinical studies have demonstrated that CAR-T cell therapy can greatly enhance patient remission and survival rates (143). Research has shown that CAR-T cell therapy can lead to an objective remission rate of 50%-70% in non-Hodgkin’s lymphoma patients, with some experiencing long-term remission. CAR-T cell therapy offers new hope for multiple myeloma patients by targeting specific antigens on myeloma cells, effectively destroying them and enhancing the patient’s health. Currently, a range of CAR-T cell therapies aimed at different blood-related cancers are in ongoing development and clinical testing, seeking to boost treatment success and lower the risk of negative reactions (144). In the coming years, CAR-T cell therapy is likely to emerge as a significant approach for managing hematological cancers. At the same time, as technology keeps progressing, CAR-T cell therapy is also being applied to treat solid tumors. For instance, certain research efforts have tried to integrate CAR-T cell therapy with ICIs to alleviate immune suppression within the tumor microenvironment and boost the antitumor effectiveness of CAR-T cells. These investigations open up new possibilities and potential for using CAR-T cell therapy in the treatment of solid tumors (145). CRS, or cytokine release syndrome, is a severe reaction that might occur during immunotherapy, notably in adoptive cell immunotherapy. A significant release of cytokines occurs when a large number of immune cells are quickly activated. The large buildup of these cytokines leads to widespread inflammatory responses, including symptoms like high fever, low blood pressure, and rapid heart rate (146). In extreme situations, it may cause respiratory failure, shock, and multiple organ dysfunction, putting the patient’s life at risk and affecting the immunotherapy process and treatment results. However, during treatment, reinfused immune cells may trigger cytokine release syndrome (CRS); approximately 60-80% of patients undergoing CAR-T therapy for ALL experience CRS to varying degrees (142). In an observational study of 50 patients receiving CAR-T therapy, approximately 30% developed mild CRS, primarily presenting with low-grade fever and fatigue that was alleviated with supportive care. Approximately 20% of patients experience moderate CRS, characterized by fever, hypotension, and tachycardia, requiring medical intervention and close monitoring (142). Approximately 10% of patients develop severe CRS with life-threatening hypotension and respiratory failure, necessitating immediate admission to the intensive care unit for emergency treatment. During CRS, many cytokines are released, some of which negatively affect the metabolism and function of skeletal muscle. For example, IL-6 activates the ubiquitin-proteasome system in skeletal muscle cells, accelerating muscle protein degradation and leading to the loss of muscle mass loss. IFN-γ can inhibit respiratory chain complex activity in the mitochondria, reducing ATP production and resulting in muscle fatigue and weakness (142). Before administering adoptive cell immunotherapy, comprehensive assessment of the patient’s physical condition, cardiopulmonary function, and muscle function is essential. Patients with pre-existing muscle disorders or functional impairments require careful risk-benefit analysis before proceeding with treatment. During therapy, close monitoring of serum cytokine levels (such as IL-6, IFN-γ, and TNF-α) should be conducted daily, along with regular muscle strength assessments (e.g., grip strength and lower limb push strength) and endurance evaluations (e.g., the six-minute walk test). Additionally, tracking the symptoms of muscle pain and fatigue is crucial. If CRS occurs and affects skeletal muscle function, its severity should be classified and appropriate interventions should be implemented. Mild CRS (temperature < 38°C, no organ dysfunction) requires supportive care, fluid and electrolyte supplementation, and close monitoring of the disease progression. Moderate CRS (temperature 38°C-39°C, mild organ dysfunction) requires supportive care combined with low-dose corticosteroids, such as dexamethasone (147). Severe CRS (temperature >39°C, severe organ dysfunction) requires the immediate administration of high-dose corticosteroids (e.g., methylprednisolone) and cytokine antagonists (e.g., tocilizumab). Conducting a thorough and systematic evaluation of the patient is crucial before starting adoptive cell immunotherapy. Assessing the patient’s basic physical state and cardiopulmonary function is essential, along with evaluating muscle function. A detailed analysis must be conducted for patients with pre-existing muscle disorders or functional impairments. Some studies are currently trying to reduce the risk of CRS by using cytokine antagonists as a preventive strategy (148). In certain clinical trials, administering monoclonal antibodies like tocilizumab before treatment can successfully inhibit IL-6 receptor signaling, thereby decreasing the occurrence and intensity of CRS. When CRS happens, it must be promptly assessed based on its severity. For mild cases, supportive care and close monitoring of disease progression are essential, and early moderate muscle activity might be considered (149). For moderate CRS, physical therapy techniques can be implemented alongside supportive care and low-dose corticosteroids. For instance, using hot compresses and massages can alleviate muscle pain and tiredness while enhancing blood flow and muscle metabolism. For severe CRS, active muscle rehabilitation therapy should accompany the use of high-dose corticosteroids and cytokine antagonists. Once the patient’s condition is stable, gradually implementing progressive resistance training and aerobic exercise can aid in restoring muscle function. Throughout rehabilitation, it’s crucial to keep a close eye on the patient’s muscle strength, endurance, and physical function metrics, and to promptly modify the rehabilitation plan based on their recovery progress (150). Concurrently, muscle rehabilitation programs such as progressive resistance training and aerobic exercise should be implemented to facilitate muscle function recovery (151).

### 4.3 Cancer vaccine

Cancer vaccines aim to stimulate the body’s specific antitumor immune response by introducing tumor-associated antigens to activate the immune system (**Figure 5C**). The HPV vaccine, known for its strong efficacy in preventing cervical cancer, continues to be a focus of research and development. Researchers are examining new HPV vaccine formulations and regimens, including long-acting protection mechanisms to decrease the number of doses required (152). Vaccination approaches for different age demographics and those with distinct immune profiles are being improved concurrently to increase the vaccine’s effectiveness and universality. From a clinical perspective, the HPV vaccine is expected to be increasingly important in the prevention of other cancers related to HPV, such as anal and oropharyngeal cancers. As awareness of the HPV vaccine increases and its coverage broadens, its role in preventing related cancers will be more clearly demonstrated worldwide (153).Clinical trials have shown that the gp100 peptide vaccine for melanoma has some anti-tumor activity, but challenges remain. Scientists are striving to enhance it in terms of research and development. For instance, they are integrating it with other immunotherapy drugs to improve the immune response. Combining the gp100 peptide vaccine with ICIs is anticipated to disrupt the tumor’s immune evasion and enhance treatment outcomes. Additionally, the use of genetic engineering technology is improving the antigen design of vaccines, enabling a more precise activation of the immune system against tumor cells. If the present challenges are addressed, therapeutic cancer vaccines could become a highly promising treatment option for those with advanced melanoma in clinical settings. These vaccines are vital components of a complete treatment plan, contributing to longer survival and better quality of life for patients (154). The success of the HPV vaccine in preventing cervical cancer serves as a model for cancer vaccine application (155). A large-scale population study with a long-term follow-up of thousands of women found that HPV vaccination significantly reduced the incidence of cervical cancer by approximately 80%. Several therapeutic vaccines have been actively explored in clinical trials for cancer treatment (156). For example, the gp100 peptide vaccine for melanoma has demonstrated antitumor activity in clinical trials, inducing specific T-cell responses in melanoma patients to inhibit tumor growth. In a clinical trial involving 50 patients with melanoma, approximately 30% of patients experienced tumor shrinkage after receiving the gp100 peptide vaccine, with 10% showing a reduction of more than 30% in tumor size. However, some patients may develop muscle fatigue, soreness, and other discomforts after vaccination. Clinical trials have reported that approximately 25% of patients experience such symptoms, usually appearing 1-3 days post-vaccination and lasting 3-7 days (157). These effects are believed to be related to the indirect impact of vaccine-induced immune responses on skeletal muscle (158). Upon immune system activation, immune cells release cytokines and immune mediators, which may affect the energy metabolism and ion balance of muscle cells. For instance, TNF-α can alter the sodium-potassium pump activity in muscle cells, leading to abnormal ion concentrations and causing muscle soreness, whereas IFN-γ can inhibit key enzymes in the glycolytic pathway of muscle cells, reducing ATP production and resulting in muscle fatigue. Further research is required to investigate the relationship between cancer vaccines and skeletal muscle function. On one hand Advanced technologies, such as single-cell sequencing and proteomics, can be used to analyze molecular changes in muscle cells induced by vaccine immune responses and to identify key signaling pathways and molecular targets (159). In an experiment using single-cell sequencing to study the effects of cancer vaccines on muscle cells, significant changes in gene expression related to these responses were observed, with the upregulation of genes associated with inflammation and downregulation of genes involved in energy metabolism, potentially correlating with muscle fatigue and soreness. Based on these research findings, vaccine design can be optimized by selecting tumor-associated antigens with higher immunogenicity and specificity, using novel delivery systems such as nanotechnology to improve antigen presentation efficiency, and adjusting vaccine dosage and administration intervals to enhance antitumor efficacy while minimizing adverse effects on skeletal muscles (160).

### 4.4 Combined chemotherapy and immunotherapy

Combined chemotherapy and immunotherapy is a highly regarded strategy in cancer treatment that aims to integrate the direct cytotoxic effects of chemotherapy on tumor cells with the immunomodulatory effects of immunotherapy to achieve synergistic enhancement and improve therapeutic outcomes (**Figure 5D**). However, this combination therapy involves complex mechanisms that require comprehensive assessment of multiple factors during implementation (161). From a mechanistic perspective, certain chemotherapeutic agents possess unique properties that enhance the efficacy of immunotherapy. For example, oxaliplatin and cyclophosphamide can induce immunogenic cell death (ICD) in tumor cells, prompting them to release tumor-associated antigens and damage-associated molecular patterns (DAMPs) (162). These molecules attract and activate immune cells, promote their infiltration into tumor tissues, and enhance the ability of immunotherapy to recognize and attack tumor cells.

A study in tumor-bearing mice demonstrated that tumor growth was significantly suppressed in the combination therapy group (chemotherapy and immunotherapy) (163). By day 14 of treatment, tumor volume was reduced by approximately 50% compared to that in the immunotherapy-only group, and immune cell infiltration in tumor tissues was notably increased, providing strong evidence that chemotherapy can amplify the immune response to inhibit tumor growth more effectively; however, combination therapy also presents challenges (164). Given these challenges, the development of personalized combination therapy regimens is crucial. First, comprehensive patient assessment should be conducted, including age, physical condition, underlying diseases (such as diabetes and cardiovascular diseases), tumor type, and stage (165). For elderly patients or those in poor physical condition, considering their lower treatment tolerance, chemotherapy doses should be appropriately reduced, less toxic immunotherapy agents should be selected, or the treatment sequence should be adjusted to minimize adverse effects. A study on elderly patients with lung cancer compared standard-dose combination therapy with low-dose chemotherapy and immunotherapy (166). The results showed that patients in the low-dose chemotherapy and immunotherapy groups had significantly fewer adverse reactions, whereas survival rates were comparable to those in the standard-dose group, highlighting the importance of personalized treatment adjustments. Second, treatment combinations should be selected based on tumor type and characteristics (11). ICIs, alone or in combination with targeted therapy, often yield optimal results for melanoma. In contrast, for lung cancer, treatment should be tailored based on PD-L1 expression levels. Patients with high PD-L1 expression may benefit more from immune checkpoint inhibitor monotherapy or combination with chemotherapy (167). For example, in non-small cell lung cancer (NSCLC), pembrolizumab (PD-1 inhibitor) combined with chemotherapy has been shown to significantly improves progression-free survival (PFS) and overall survival (OS) in non-small cell lung cancer (NSCLC). This is because high PD-L1 expression allows tumor cells to evade immune surveillance more effectively, whereas pembrolizumab blocks the PD-1/PD-L1 pathway, restoring immune system activity against tumor cells (168). Combined chemotherapy then directly kills tumor cells, achieving a synergistic therapeutic effect, and the sequence of drug administration plays a key role in optimizing the therapeutic outcomes.

For some solid tumors, administering chemotherapy first to induce the release of tumor antigens, followed by immunotherapy, can enhance the ability of immune cells to recognize and attack tumor cells, thereby activating the immune system more effectively (169). In contrast, for certain hematologic malignancies, simultaneous administration of chemotherapy and immunotherapy may yield superior results. In lymphoma treatment, concurrent use of chemotherapy and immunotherapy has been shown to enhance tumor clearance rates and improve response durability (170). This may be due to the rapid growth and proliferation of hematologic tumor cells, making the simultaneous administration of both therapies more effective in suppressing tumor progression. Furthermore, immunotherapy can increase tumor sensitivity to chemotherapy, further improving the treatment efficacy. By precisely adjusting treatment parameters, combined chemotherapy and immunotherapy can maximize their complementary advantages, enhance antitumor efficacy while minimizing skeletal muscle damage, and improve quality of life and treatment tolerance, ultimately offering better therapeutic prospects for cancer patients (171).

## 5 Limitation of immunotherapy and future prospect

Immunotherapy has made significant progress in the field of cancer treatment and has brought new hope to many cancer patients; however, it also has several limitations. For example, immunotherapy can have negative effects on skeletal muscles by disrupting normal function. ICIs can cause muscle-related symptoms in approximately 30% of patients, including muscle pain, which often affects proximal limb muscles such as the shoulders and hips, with varying pain intensities (172). Additionally, muscle weakness is common and significantly affects daily activities and the quality of life. Adoptive cell immunotherapy may trigger cytokine release syndrome (CRS), with 60-80% of patients undergoing CAR-T therapy for acute lymphoblastic leukemia (ALL) experiencing CRS to varying degrees. During CRS, cytokines such as IL-6 activate the ubiquitin-proteasome system in skeletal muscle cells, accelerating muscle protein degradation and leading to muscle mass loss. IFN-γ inhibits the mitochondrial respiratory chain complex activity, reducing ATP production, which results in muscle fatigue and weakness. Cancer vaccines may also cause muscle-related side effects in some patients (173). Approximately 25% of vaccine recipients experience muscle fatigue and soreness, typically appearing 1-3 days post-vaccination and lasting 3-7 days. These effects are believed to be linked to immune responses that affect energy metabolism and ion balance of muscle cells. For instance, TNF-α alters Na+/K+ pump activity, leading to ion imbalance and muscle soreness, whereas IFN-γ suppresses key glycolytic enzymes, reducing ATP production, and causing muscle fatigue. The efficacy of immunotherapy varies significantly among patients and is influenced by factors, such as tumor microenvironment complexity, genetic background, and immune system status. For instance, in multiple myeloma, interactions between tumor cells and immune cells in the tumor microenvironment affect disease progression and the response to immunotherapy. Differences in immune cell infiltration and function among patients make it difficult to standardize treatment plans, thereby increasing the clinical challenges. Immunotherapy may also cause various immune-related adverse effects (irAEs) beyond its impact on skeletal muscles, affecting multiple organ systems (174). Excessive immune activation can trigger a cytokine storm, leading to a massive release of cytokines such as IFN-γ, TNF-α, and IL-6, which not only indirectly damages skeletal muscle cells but also elevates systemic inflammatory cytokine levels, induces protein degradation, inhibits protein synthesis, and impairs muscle fiber regeneration and repair, ultimately forming a vicious cycle. Furthermore, immunotherapy may cause or exacerbate autoimmune diseases. For instance, ICIs have been linked to thyroid dysfunction, pneumonia, and other complications, affecting overall patient health and increasing treatment risks and complexities (175). Some patients develop resistance to immunotherapy over time, leading to diminished efficacy or treatment failure. Resistance to ICIs has been observed in cancers such as melanoma and lung cancer, where tumor cells can evade immune system attacks through mechanisms such as upregulation of immunosuppressive molecules or alteration of the tumor microenvironment, ultimately limiting the long-term effectiveness of immunotherapy and posing significant clinical challenges in overcoming resistance. Research in the future could address immunotherapy resistance by focusing on three key aspects. To start, devise innovative combination therapies. Look into more precise combinations of immunotherapy with targeted therapy, like integrating drugs based on specific gene mutations in tumors (176). Also, the integration of immunotherapy with novel technologies like oncolytic virotherapy promises to enhance treatment effectiveness in melanoma. Secondly, boost initiatives to discover new targets through the use of advanced technologies for analyzing the molecular features of tumor and immune cells. Create medications targeting new areas such as tumor-specific glycoproteins or molecules that regulate the immune system, which can prevent tumors from evading the immune system and boost the immune response against tumors. Thirdly, investigate tumor heterogeneity through multi-omics analysis to grasp its connection with immunotherapy resistance. Choose tailored immunotherapy medications and combinations according to the specific traits of a person’s tumor (177). In breast cancer, for instance, personalized treatments can enhance patient survival and provide novel methods to combat resistance. Moreover, the high cost of immunotherapy imposes a significant financial burden on patients and the healthcare system (178). The cost of certain novel ICIs and CAR-T cell therapies ranges from hundreds of thousands to millions of yuan, making them unaffordable for many patients and limiting their widespread clinical application. Moreover, immunotherapy often requires long-term administration, further increasing treatment costs and posing serious challenges to the allocation of healthcare resources (179).

## 6 Conclusion

Our work provides an in-depth exploration of the complex interactions within the tumor microenvironment, focusing on the formation of immune niches, their underlying mechanisms, and therapeutic potential. There are intricate interactions between immunotherapy, the tumor microenvironment, and the biomechanics of skeletal muscle. Despite its effectiveness in cancer treatment, immunotherapy adversely affects skeletal muscle. Muscle-related symptoms can occur in about 30% of patients treated with ICIs (180). Muscle discomfort is a potential side effect of cancer vaccines in some patients. Also, alterations in the tumor microenvironment influence skeletal muscle metabolism and function through various pathways. The tumor microenvironment is a dynamic ecosystem in which immune cells, tumor cells, and stromal cells interact, forming distinct immune niches (181). These niches play a critical role in shaping the immune response against tumors and are key factors in the success of immunotherapy. A thorough exploration of the mechanisms behind immune niche formation can aid in creating more effective immunotherapy strategies. For instance, improving anti-tumor immune responses by aiming at specific parts of immune niches or merging different immunotherapy methods to surpass the restrictions imposed by immune niches (182). Natural killer (NK) cells, macrophages, dendritic cells, and T cells are major participants in the tumor microenvironment. NK cells release perforin and granzymes to kill tumor cells, whereas macrophages can polarize into M1 or M2 phenotypes. M1 macrophages exhibit proinflammatory and antitumor properties, whereas M2 macrophages often promote tumor growth. Dendritic cells are key mediators of antigen presentation, activating T cells to recognize and attack tumor cells. Additionally, regulatory T cells (Tregs) and myeloid-derived suppressor cells (MDSCs) produce immunosuppressive cytokines, inhibit immune responses, and facilitate tumor immune evasion (21). This intricate network of interactions contributes to the formation of unique immune niches with distinct immunological characteristics. Several factors can influence the formation of immune niches: cancer-associated fibroblasts (CAFs) release extracellular matrix components, modifying the physical and chemical properties of the microenvironment, thereby influencing immune cell infiltration and function (183). Tumor-derived factors, such as cytokines and chemokines, recruit immune cells to specific locations and shape the localized immune microenvironment. Moreover, genetic and epigenetic changes in tumor cells regulate their interactions with immune cells, further sculpting immune niches. Understanding the mechanisms underlying the immune niche formation has important therapeutic implications. Immunotherapies, such as ICIs, adoptive cell therapy, and cancer vaccines, aim to modulate the immune system to target tumors. However, their efficacy is closely linked to the immune niches within the tumor microenvironment. For example, ICIs block inhibitory receptors on immune cells; however, their effectiveness may be limited by immunosuppressive immune niches (184). Similarly, adoptive cell therapies, such as CAR-T cell therapy, may rely on the tumor microenvironment’s capacity to support the survival and function of transferred immune cells. Cancer vaccines designed to stimulate immune responses against tumor-associated antigens may also face challenges in terms of immunosuppressive niches. By conducting comprehensive research on immune niche formation in the tumor microenvironment, we can develop more effective immunotherapy strategies. This includes targeting specific components of immune niches to enhance antitumor immune responses, such as blocking immunosuppressive signals or promoting recruitment and activation of antitumor immune cells. Combining different immunotherapies or integrating immunotherapy with other treatment modalities (e.g., chemotherapy) may help overcome the limitations imposed by immune niches and ultimately improve patient outcomes (185). In conclusion, research on immune niche formation within the tumor microenvironment is a rapidly evolving field with tremendous potential for improving cancer treatment. Additional research is required to fully understand the complex mechanisms at play and to apply these insights to develop more effective clinical therapies, ultimately improving cancer patient survival and quality of life (186).
